# Shaping metallic glasses by electromagnetic pulsing

**DOI:** 10.1038/ncomms10576

**Published:** 2016-02-08

**Authors:** Georg Kaltenboeck, Marios D. Demetriou, Scott Roberts, William L. Johnson

**Affiliations:** 1 Keck Engineering Laboratories, California Institute of Technology, Pasadena, California 91125, USA

## Abstract

With damage tolerance rivalling advanced engineering alloys and thermoplastic forming capabilities analogous to conventional plastics, metallic glasses are emerging as a modern engineering material. Here, we take advantage of their unique electrical and rheological properties along with the classic Lorentz force concept to demonstrate that electromagnetic coupling of electric current and a magnetic field can thermoplastically shape a metallic glass without conventional heating sources or applied mechanical forces. Specifically, we identify a process window where application of an electric current pulse in the presence of a normally directed magnetic field can ohmically heat a metallic glass to a softened state, while simultaneously inducing a large enough magnetic body force to plastically shape it. The heating and shaping is performed on millisecond timescales, effectively bypassing crystallization producing fully amorphous-shaped parts. This electromagnetic forming approach lays the groundwork for a versatile, time- and energy-efficient manufacturing platform for ultrastrong metals.

## Abstract (web summary)

Metallic glasses can be softened to different shapes whilst maintaining mechanical properties, and the related thermoplastic processing requires a heating source and an applied force. Here, the authors show an effective thermoplastic approach by a coupling between electric pulses and a magnetic field.

## Graphical abstract

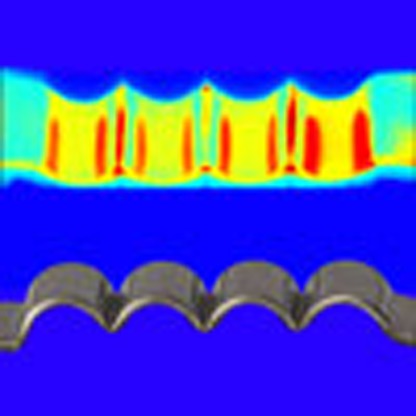

The concept of a Lorentz force generated on a current-carrying conductor exposed to a magnetic field dates back to the nineteenth century work of Faraday and Maxwell on electromagnetism^1^. In this concept, moving point charges comprising the electric current experience Lorentz forces, which consist of electric and magnetic force components. The magnetic point forces combine to produce a magnetic body force, often referred to as the Laplace force, acting on the current-carrying conductor. If the conductor is a metallic glass, the Laplace force provides for innovative methods of forming. Owing to unique electrical resistivities, metallic glasses can be rapidly and uniformly heated when electrical energy is dissipated in them^2^,^3^,^4^. Combining ohmic dissipation with the application of Laplace force creates a powerful platform to process metallic glasses.

While metallic glasses are generally known for their attractive mechanical properties^5^,^6^, perhaps their most promising attribute is their potential for ‘thermoplastic' processing^7^,^8^,^9^,^10^,^11^,^12^,^13^,^14^. By virtue of being glasses, they can be softened to viscous liquid states above the glass transition where viscoplastic shaping can be carried out in a manner similar to that applied to process conventional thermoplastics. This prospect paved the way to nano-fabrication and nano-moulding, revealing a remarkable ability to replicate nano-features down to 10 nm length scales^10^,^11^. Unfortunately, this potential for thermoplastic forming is practically limited by the rapidly intervening crystallization of the relaxed ‘supercooled' liquid. Electrical discharge heating has recently emerged as an effective means to overcome this limitation^2^,^3^,^4^. It enables rapid and spatially uniform heating to low-viscosity states considered to be optimal for thermoplastic shaping, over timescales sufficiently short to bypass crystallization.

In this work, we demonstrate that subjecting the metallic glass to an intense electric current pulse directed normal to an applied magnetic field can generate Laplace forces sufficiently large to perform thermoplastic shaping operations thereby producing high-quality net-shaped metallic glass articles.

Electromagnetic forming of conventional (that is, crystalline) metals has been explored since the late 1950s (refs 15, 16). In the most widely used approach, an induction coil excited by a current pulse generates a transient magnetic field that induces eddy currents in a workpiece according to Faraday's law of induction. The coupling of the eddy currents and the magnetic field produces a repulsive Lorentz force, in accordance with Lenz's law, which accelerates the workpiece away from the restrained coil and against a tool causing it to form. This approach results in high workpiece velocities (typically >100 m s^−1^) and strain rates on the order of 10^4^ s^−1^ during formation^16^,^17^. Although some heat may be generated in the sample by induction, the forming process takes place entirely in the solid (that is, the crystalline) state. Generally, the generated Lorentz forces must be high enough to produce an equivalent impact pressure (typically hundreds of MPa) that exceeds the material yield strength, thereby enabling large plastic deformation. The metallic sample must therefore have high electrical conductivity, low yield strength and also be of limited thickness. Because of these requirements, electromagnetic forming of metals has so far been limited mostly to aluminium and copper in thin sheet and tube geometries, while stronger, less conductive metals such as steels have been largely excluded.

Compared with crystalline metals, amorphous metals demonstrate considerably higher room-temperature yield strengths (1–2 orders of magnitude higher than copper and aluminium)^5^,^6^. However, in their relaxed viscous state above the glass transition, the flow stresses are much lower (on the order of tens of MPa or less at temperatures substantially higher than the glass transition)^18^,^19^,^20^, and the associated viscosities within the range where conventional plastics are typically processed (that is, 10^0^–10^4^ Pa s)^21^,^22^. Processing a metallic glass in this optimum rheological window is limited by crystallization of the supercooled liquid, which typically limits the available processing time to under a second. Hence, a rapid forming approach for metallic glasses that utilizes an intense electromagnetic pulse to thermoplastically shape a bulk metallic glass in its relaxed viscous state would be attractive, as it would require much lower forming stresses than conventional aluminium and copper while final metallic glass parts would be considerably stronger.

Another fundamental distinction between crystalline and amorphous metals is their different electrical resistivities. Most amorphous metals exhibit electrical resistivities in the range of 150–200 μΩ cm (ref. 23), which are much larger than those of crystalline metals (typically 1–50 μΩ cm). Furthermore, unlike crystalline metals, the electrical resistivity of amorphous metals is nearly constant or even decreasing with temperature^24^. The large and temperature independent electrical resistivity hinders generation of large magnetic pressures during conventional electromagnetic forming; however, it enables efficient and stable volumetric heating by uniform ohmic dissipation. Indeed, it has been shown that ohmic heating of metallic glasses can be both rapid and uniform, enabling thermoplastic processing at temperatures far above the glass transition^2^,^3^,^4^. In this work, we demonstrate that the electrical and rheological properties of metallic glasses are such that a window exists for an electromagnetic forming process. Specifically, we show that by coupling a uniform electric current with an applied static magnetic field, one can simultaneously bring a metallic glass to a low-viscosity state while exploiting the accompanying Laplace force to form the sample to a net shape, all while avoiding crystallization.

## Results

### Identifying a process window

**Figure 1:**
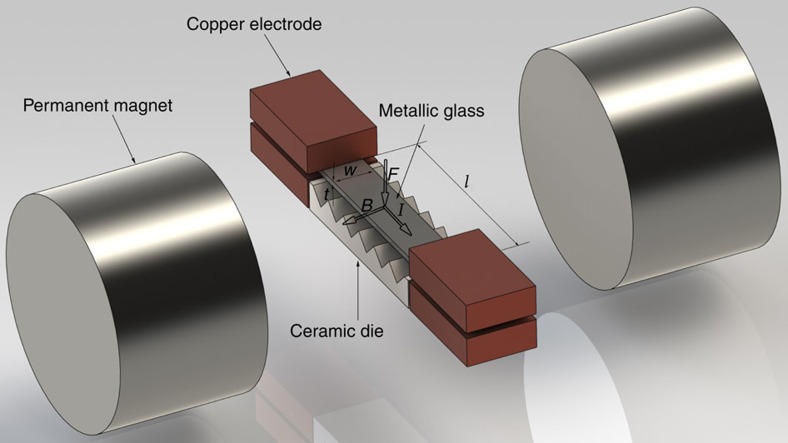
Schematic representation of the electromagnetic forming configuration. The schematic presents a strip of metallic glass having length *l*, width *w* and thickness *t*, subject to traversing electrical and magnetic fields. The strip is in contact with copper electrodes delivering a current *I* along *l*, and normal to a pair of permanents magnets inducing a magnetic field *B*. A Laplace force *F* is shown to be generated on the strip normal to the electric and magnetic fields (according to a ‘left-hand rule'). A ceramic die is also shown placed at the side of the strip opposite to the direction of *F*.

To process metallic glasses thermoplastically by electromagnetic forming, it is essential that the processes of ohmic heating and magnetic forming are independently controlled. This requires that the electric current, which travels through the metallic glass dissipating electrical energy and producing volumetric heating, be controlled independently of the magnetic field, which interacts with the electric current to generate the magnetic force. This is achieved by placing the metallic glass in series with an electric current source directed transverse to an independently applied magnetic field. Such configuration is shown in Fig. 1, where a metallic glass sample is connected in series to a capacitor via electrodes while situated transverse to a magnetic field generated by two permanent magnets. Application of an intense electric current pulse ohmically heats the metallic glass rapidly and uniformly to a predetermined temperature in the supercooled liquid region, while the coupling between electric current and magnetic field generates a force pulse on the sample urging it against a permanent die tool, where it forms and subsequently cools and revitrifies. Here, we show that typical currents produced by discharge of a conventional capacitor and typical magnetic field strengths generated by conventional permanent magnets are adequate to rapidly and uniformly heat a bulk metallic glass sample to a low-viscosity state and thermoplastically shape it against a die tool before the intervention of crystallization.

The Laplace force on a current-carrying conductor exposed to a magnetic field is given by 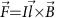, where *I* is the current travelling through the conductor, 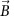 is the magnetic field, and 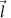 is a conductor length vector along the current direction that traverses the magnetic field. When the magnetic field is normal to 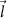, the magnitude of the Laplace force reaches a maximum given by *F*=*BIl*, while any non-parallel configuration of 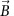 and 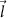 produces a fraction of this maximum value. For a typical metallic glass, one can show that the time-average current *I* discharged on a time constant *τ* required to ohmically heat a sample to a temperature in the supercooled liquid region where the viscosity is in the order of ∼10^2^ Pa s is approximately (see Methods)^23^,^25^,^26^:


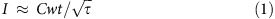


where *t* is the sample thickness in the direction of the Laplace force, and *C* is a constant involving the material thermal and electrical properties, which for most metallic glasses ranges between 2 and 4 × 10^7^ A √s m^−2^ (see Methods)^27^,^28^,^29^,^30^. The magnitude of the applied Laplace force is then


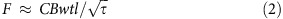


where *w* is the conductor projected width along the direction of the magnetic field. The pressure *P* exerted by the applied Laplace force is


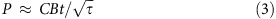


The Laplace force according to equation 2 appears dependent on the sample volume; its magnitude, however, can be independently controlled through *B* and *τ*. Pressure on the other hand, which is the fundamental parameter determining the shaping capacity, scales with the sample thickness and is likewise controllable through *B* and *τ*.

Equations 1, 2, 3 may be thought of as semi-quantitative relations allowing one to identify a process window for implementing this approach with a metallic glass. As an example, a metallic glass sheet feedstock undergoing electromagnetic forming having *w*=*l*=100 mm and *t*=1 mm may be considered. Using equations 1, 2, 3 and assuming *B*∼1 T, typical of a set of conventional permanent magnets, and τ∼1 ms, typical of a capacitive discharge circuit, one may estimate *I*∼10^5^ A, *F*∼10 kN and *P*∼10^5^ Pa (that is, *P*∼1 atm). These are of the order of forces and pressures applied in typical blow moulding processes for plastics performed at viscosities in the range of 10^0^–10^4^ Pa s (refs 21, 22), and should be sufficient to induce substantial strain rates in a softened metallic glass heated high enough into the supercooled liquid region^18^,^19^,^20^. As the metallic glass would be ohmically heated to within this viscosity regime and the associated electromagnetic pressure would be on the order of typical blow moulding pressures, the strains produced would likewise be comparable to those achieved in thermoplastic blow moulding of plastics. Hence, in terms of general forming capacity, electromagnetic forming of a metallic glass sheet would be akin to thermoplastic blow moulding. It is also important to note that the Laplace force will always be applied normal to the sheet (in the plane normal to the magnetic field). As such, the forming pressure is effectively ‘hydrostatic', thereby mimicking the application of gas pressure in thermoplastic blow moulding.

### Implementing electromagnetic forming

Here we demonstrate this concept by subjecting Zr_35_Ti_30_Cu_7.5_Be_27.5_ metallic glass strips with *l*=33 mm, *w*=7 mm and *t*=1.0 mm to traversing electric and magnetic fields to electromagnetically form them against permanent die tools with semicircular corrugations, as illustrated in Fig. 1 (see Methods). A strip is placed between two FeNdB permanent magnets with its length *l* oriented normal to the magnetic field, while a capacitor with millisecond time constant is discharged to deliver a rapid current pulse to the strip along *l* through the contacting electrodes. A ceramic die is placed alongside the strip where deformation would be induced by the Laplace force (that is normal to the electric and magnetic fields according to a ‘left-hand rule'). A capacitive discharge circuit with 0.264 F capacitance is used, and the applied voltages ranged between 68 and 71 V. A measured magnetic field of ∼0.275 T is produced by the permanent magnets at the location of the strip. The millisecond current pulse generated by the discharging capacitor rapidly heats the metallic glass in open air to a viscous state conducive for thermoplastic forming, while the Laplace force generated by the electromagnetic interaction between electrical and magnetic fields drives the softened metallic glass against a permanent die to shape it and simultaneously cool and revitrify the sample by thermal conduction to the die.

**Figure 2:**
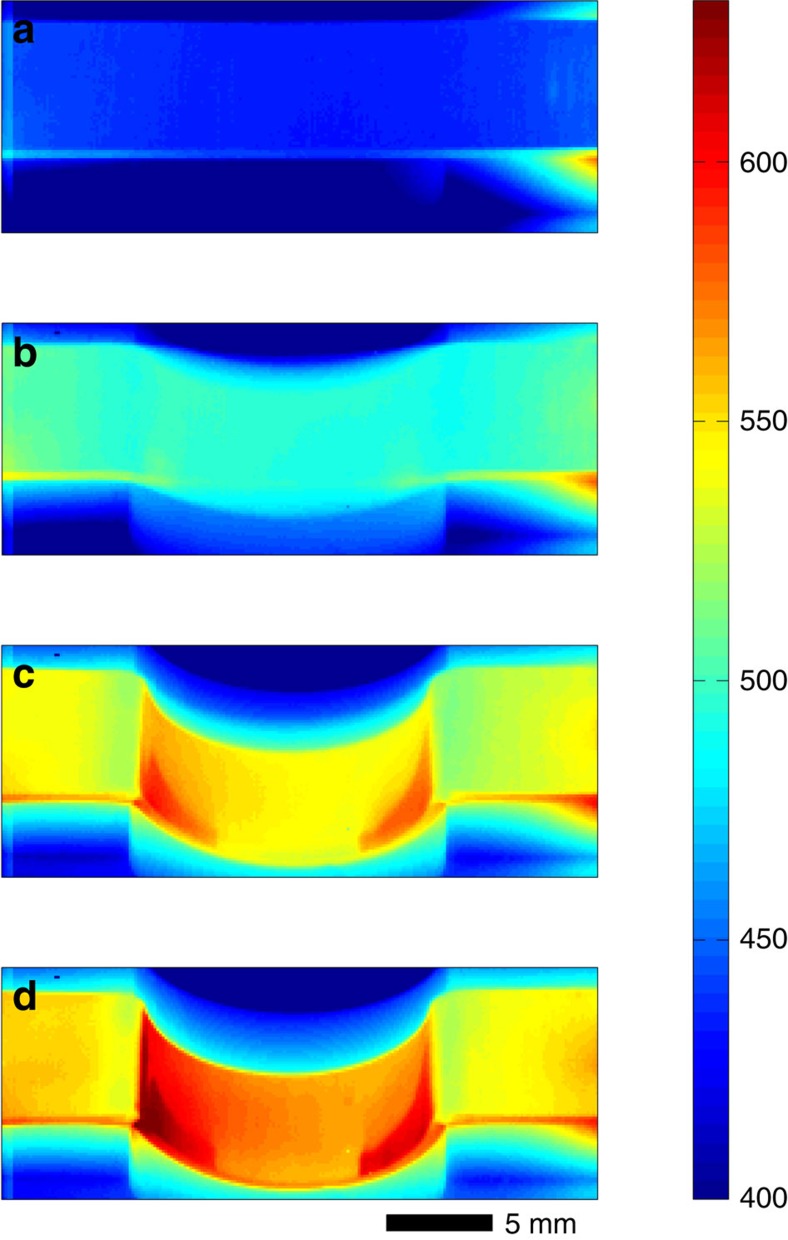
Time evolution of the temperature distribution during electromagnetic forming. (**a**) 0 ms; (**b**) 1 ms; (**c**) 2 ms; and (**d**) 3 ms. An amorphous Zr_35_Ti_30_Cu_7.5_Be_27.5_ strip is undergoing heating and deformation by electromagnetic forming, as recorded by an infrared thermal imaging camera. Deformation is terminated at about 3 ms as the strip completely forms against the die (not visible by thermal imaging).

**Figure 3:**
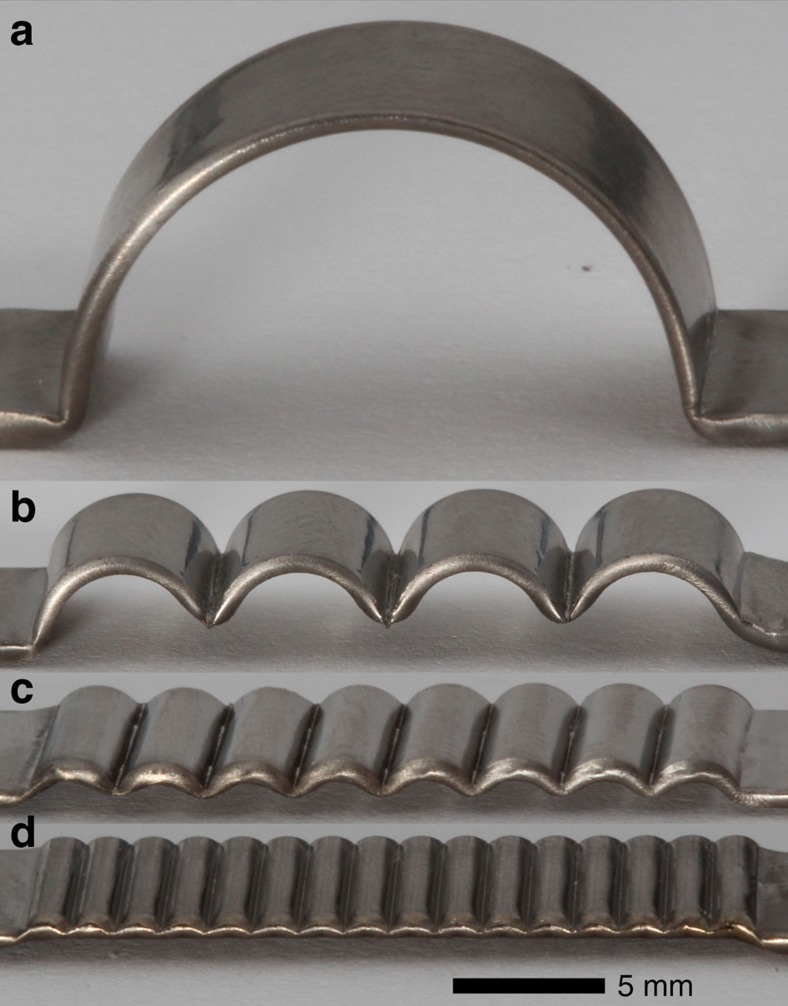
Electromagnetically formed corrugated amorphous strips. Amorphous Zr_35_Ti_30_Cu_7.5_Be_27.5_ strips are formed electromagnetically against dies of progressively finer semicircular corrugations. Images **a**–**d** show strips formed with 1, 4, 8 and 16 semicircular corrugations. Image **a** presents the formed strip shown in the thermographs of Fig. 2.

The evolution of electromagnetic forming against a die with a single semicircular corrugation is captured by a thermal imaging camera and is presented in Fig. 2. As seen in Fig. 2, the strip is heated rapidly and uniformly attaining a process temperature in the range of 500–550 °C in 1–2 ms. In this temperature range, the viscosity of Zr_35_Ti_30_Cu_7.5_Be_27.5_ is between 10 and 100 Pa s (ref. 8). As the heating appears to saturate, deformation of the strip initiates owing to the generated Laplace force. The plastically deforming strip is pressed against the ceramic die at room temperature (not visible by thermal imaging) forming a semicircular bow. The heating and forming process are completed over a time of 3–4 ms, followed by cooling and revitrification occurring over a longer time (not shown). Crystallization is entirely avoided during the ultrarapid heating and forming processes, as well as during cooling; the amorphous structure of the formed strip is verified by X-ray diffraction. The final formed amorphous strip is shown in Fig. 3a. Figure 3b–d shows three more amorphous strips formed with 4, 8 and 16 semicircular corrugations produced using the same process conditions. The formed strips demonstrate fine replication of the progressively finer corrugated dies, which is a consequence of a low and fairly uniform viscosity, and also of a uniformly and hydrostatically applied force.

### Analysis of the process parameters

**Figure 4:**
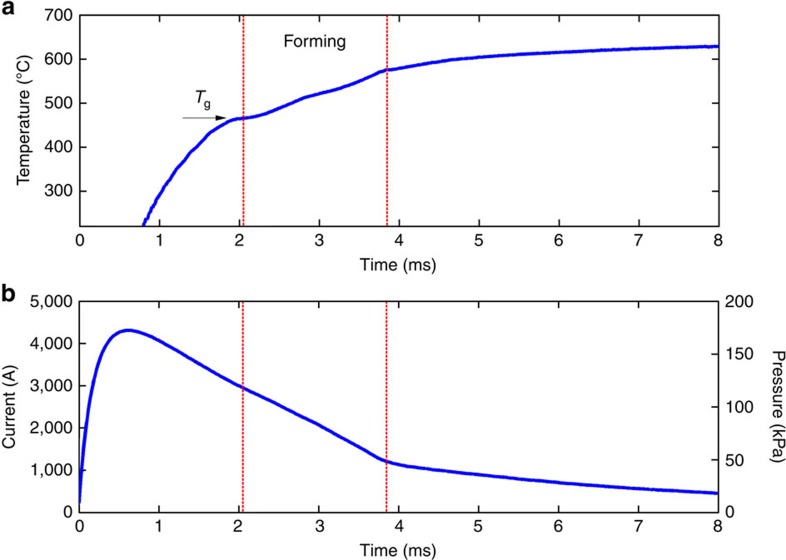
Time-dependent thermal and electrical response. (**a**) Time-dependent sample temperature, as monitored by an infrared pyrometer; (**b**) time-dependent current through the sample as measured by a Rogowiski coil (left-hand axis) and time-dependent magnetic pressure estimated by *P*=*BI*/*w* (right-hand axis), during ohmic heating and forming of the amorphous Zr_35_Ti_30_Cu_7.5_Be_27.5_ strip presented in Fig. 2. The ‘forming' time interval is indicated by vertical lines in **a**,**b** and the dynamic glass-transition temperature *T*_g_ is indicated by an arrow in **a**.

In Fig. 4, we plot the time-dependent sample temperature, as monitored by an infrared pyrometer, the current through the sample as measured by a Rogowski coil, and the applied magnetic pressure as estimated by *P*=*BI*/*w*, during the heating and deformation of the strip shown in Fig. 2 (see Methods). The time interval of the ‘forming region', where deformation of the strip initiates once a low enough viscosity is reached under an applied magnetic force and terminates when the strip is in full contact with the die, is indicated in all three plots. In Fig. 4a, the temperature is seen to rise rapidly crossing the dynamic glass transition at about 475 °C (about 170 °C above the calorimetric glass transition measured at 20 K min^−1^) at about 2 ms. The temperature continues to rise in the deformation regime between 500 and 550 °C but in a somewhat discontinuous manner, as the strain developing in the strip increases its electrical resistance which contributes to incrementally more efficient ohmic heating. The temperature equilibrates to about 600 °C at about 6 ms. The current in Fig. 4b is shown to rapidly peak at slightly above 4,000 A in just less than 1 ms, then slowly declines towards zero at large timescales. The magnetic pressure as calculated from *P*=*BI*/*w* (by assuming normal electric and magnetic fields) is plotted on the right-hand axis in Fig. 4b. With these assumptions, *P* is simply a linear superposition of *I*. The peak pressure reached in just less than 1 ms is about 170 kPa, while the pressure within the forming region declines gradually from 120 to 60 kPa N. These values are well within the range of pressures commonly used in thermoplastic blow moulding of plastics, as discussed above.

## Discussion

In the simple configuration presented here, where the current travelling through a sample is directed normal to a magnetic field induced by two permanent magnets, the magnitude of the magnetic force can be independently varied by controlling the strength of the magnetic field. But since the magnetic field in this configuration is constant, the timing of the magnetic force is solely determined by the time-dependent current, and thus is not independently controllable. As shown in Fig. 4, being proportional to the current, the force peaks before the metallic glass reaches the least viscous supercooled state. Even though the applied force is sufficient for thermoplastic forming, the timing of the force application is not ‘optimal'. More ‘optimal' timing of the process, where a constant magnetic force of a desired magnitude is applied when a desirable viscous state is reached, can be achieved by more complex electric/magnetic configurations. For instance, configurations that involve two or more successive current pulses with appropriately chosen rise times and amplitudes can achieve sequencing of the heating and forming process. Furthermore, configurations that utilize electromagnets (such as Helmholtz coils) instead of permanent magnets, where magnetic pulses with desirable profiles can be generated electrically, may achieve even more effective sequencing of the two processes.

The discussion and example above are focused on the ‘forging' or ‘stamping' of a strip or sheet using an operation mode akin to thermoplastic blow moulding. This is the most natural and straight-forward implementation of this forming approach. However, the general concept introduced here could in principle be configured to perform other thermoplastic shaping operations involving samples in bulk geometries. For example, using a different feedstock geometry and a different magnetic field configuration, one may be able to operate in an ‘injection moulding' or ‘calendaring' (sheet extrusion) mode. Specifically, using a suitable configuration of multiple permanent magnets or electromagnets disposed at different angles relative to the electric field axis, a distribution of magnetic forces can be generated on the feedstock that are capable of guiding it through a mould runner and gate and into a mould cavity of a desirable shape, or between rollers to shape it into a sheet. As feedstock, one may use a bulk prismatic sample having *l*=100 mm, *w*=10 mm and *t*=10 mm. Assuming *B*∼1 T and∼1 ms, using equations 1, 2, 3 one may then estimate the current required to ohmically heat to a viscous supercooled state as *I*∼10^5^ A, the magnetic force *F*∼10 kN, and the pressure *P*∼10^6^ Pa (that is, ∼1 MPa). While *I* and *F* are on the same order as in the sheet forming process, a pressure on the order of ∼1 MPa is near the lower bound of pressures used in thermoplastic injection moulding or calendaring of conventional plastics (refs 21, 22). As a *t* of 10 mm is near the upper bound of the achievable casting thickness of typical metallic glass formers and a *B* of 1 T is near the upper limit for conventional permanent magnets, the window for an injection moulding or calendaring process may be quite narrow.

The dependence of the Laplace force and pressure on the sample dimensions along with limitations in magnetic field strength may be seen as a drawback of the present approach when compared against conventional mechanical technologies (for example, utilizing hydraulic presses, pneumatic drives, electrical motors and so on), where larger forces and pressures are attainable. But the present concept offers unique advantages over conventional mechanical forming processes. As discussed above, the Laplace force is applied normal to the deforming sample leading to pressures that are effectively hydrostatic, unlike conventional methods where forces are generally unidirectional. Furthermore, spatially varying magnetic fields may be applied to produce varying Laplace force directions in different regions of the sample permitting complex shaping operations. From a manufacturing perspective, the absence of a working medium (that is, the lack of presses, motors, drives and massive fixtures) leads to an essentially frictionless process with minimal wear, and provides ease of automation. Moreover, because only the sample is accelerated during the process, large strains can be achieved in timescales significantly shorter than typical mechanical forming processes (that is, in milliseconds rather than tens of milliseconds; see Figs 2 and 4) such that process cycle times may be substantially reduced. Despite all these technical advantages, the net economic advantages over the incumbent technology and the overall commercial viability of this approach would be difficult to gauge at this stage.

In summary, using a simple electromagnetic setup comprising a capacitor bank and a pair of permanent magnets to subject a metallic glass to traversing electric and magnetic fields, we demonstrate that the metallic glass can be formed thermoplastically in a millisecond timescale in open air in the absence of any conventional heating source or any applied mechanical force by coupling ohmic heating and magnetic forming. This simple demonstration lays the foundation for a time and energy efficient all-electronic manufacturing platform for amorphous metals that could rival the simplicity and economics of plastics manufacturing.

## Methods

### Sample preparation

Amorphous Zr_35_Ti_30_Cu_7.5_Be_27.5_ strips 1 mm in thickness, 7 mm in width and 4 cm in length were prepared by arc-melting the elemental constituents in a water-cooled copper hearth under a titanium-gettered argon atmosphere, followed by suction casting in a copper mould. The strips were ground flat and parallel, and their amorphous structure was verified by X-ray diffraction. Corrugated dies used for the shaping process were machined from MACOR ceramic.

### Electromagnetic setup configuration

The electromagnetic forming setup comprises a capacitor bank with capacitance of 0.264 F rated for voltage of up to 100 V, a set of copper clamps connected to the capacitor bank via copper leads that hold the metallic glass sample and deliver the current discharge across it. Two NdFeB cylindrical magnets (3′ in diameter and 2′ in width) held on either side of the sample to create a magnetic field perpendicular to the current flow and parallel to the die's moulding surface. The setup operates in open air. The magnetic field strength in the vicinity of the sample is measured using a hall-probe Gaussmeter to be 0.275 T. Also, the time-dependent current through the sample during the electrical discharge was measured using a Rogowski coil wound around a current-carrying lead.

### Thermal imaging

A high-speed infrared imaging camera (FLIR Corp., SC2500) with a spectral band from 0.9 to 1.7 mm outfitted with a band-pass filter allowing wavelengths from 1.5 to 1.9 mm was employed to record the evolution of the temperature distribution and deformation at frame rates in the range of 994–1,500 frames s^−1^. An IMPAC IGA740-LO high-speed infrared pyrometer with a spectral band from 1.58 to 2.2 mm and a response time of 6 μs was also used to record temperature vs. time in a circular focal spot of 1 mm diameter in the section of the strip being formed. Both camera and pyrometer were calibrated simultaneously by tracking the melting of Pd_43_Ni_10_Cu_27_P_20_ alloy with a solidus temperature of 531 °C, which is within the temperature range considered in this work. Using this method, emissivities of 0.285 and 0.26 were found for the infrared camera and pyrometer, respectively. The emissivities are expected to be roughly representative of Zr_35_Ti_30_Cu_7.5_Be_27.5_ liquid in the temperature range of 450–600 °C, and the temperature error is expected to be within 20 °C.

### Analysis of Ohmic heating

The electrical energy *E* dissipated to ohmically heat a prismatic metallic glass sample having length *l* along the direction of current flow, width *w* and thickness *t* from an initial temperature *T*_o_ (typically room temperature) to a process temperature *T* in the supercooled liquid region (that is, above the glass-transition temperature *T*_g_) where the viscosity is on the order of 10^2^ Pa s is given by:


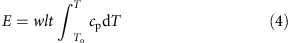


where *c*_p_ is the specific heat capacity of the material in J m^−3^ K^−1^. Assuming that *c*_p_ of the glass and the supercooled liquid is a constant (that is, independent of temperature) as the material is heated through *T*_g_, equation 4 can be approximated as


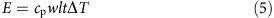


where Δ*T* is the temperature rise from *T*_o_ to *T*. For most metallic glasses,


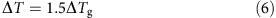


where Δ*T*_g_=*T*_g_–*T*_o_ (ref. 25). If one approximates the time-dependence of a current pulse produced in an electrical discharge having a characteristic discharge time *τ* by an isosceles triangle, the electrical energy dissipated during the discharge can be estimated as:


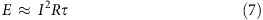


where *I* is the peak current attained at time *τ*, and *R* is the electrical resistance of the metallic glass sample, given by


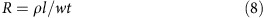


where *ρ* is the electrical resistivity of the metallic glass. Substituting equations 6, 7, 8 into equation 4 and solving for *I*, one can obtain the electrical current required to ohmically heat a metallic glass sample a temperature where the viscosity is on the order of 10^2^ Pa s as follows:


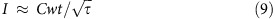


The constant *C* has units of A √s m^−2^, and involves the material properties as follows:


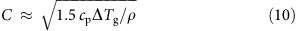


Here we estimate the constant *C* for a wide variety of metallic glass compositions. The temperature-dependent resistivity of metallic glasses is known to vary over a narrow range, typically from 150 to 200 μΩ cm (ref. 23). For the sake of simplicity, we approximate the resistivity of all metallic glasses by a mean value of 175 μΩ cm (1.75 × 10^−6^ Ω m). Moreover, an average specific heat capacity between room temperature and 1.5Δ*T_g_* will be assumed for *c*_p_. In this temperature range, the specific heat capacity in J mol^−1^ K^−1^ for most metallic glass compositions as they are heated through the glass transition varies between 25 and 45J/mol^−1^ K^−1^; here we assume a mean value of ∼35 J mol^−1^ K^−1^ (ref. 26). But the specific heat capacity in J m^−3^ K^−1^, as used in equations 4 and 10, can vary substantially between metallic glass compositions because of the variation in the molar volume between compositions. Here we estimate the specific heat capacity in J m^−3^ K^−1^ for each metallic glass composition as the ratio between 35 J mol^−1^ K^−1^ and molar volume. Lastly, Δ*T*_g_, estimated here as Δ*T*_g_=*T*_g_–298 K, also varies considerably between metallic glass compositions.

In Table 1 we present the values for Δ*T*_g_, molar volume *v*_m_ and *c*_p_ in J m^−3^ K^−1^ (estimated as the ratio between 35 J mol^−1^ K^−1^ and *v*_m_) for a Au-based, Pt-based, Pd-based, Zr-based and Fe-based metallic glass to estimate the constant *C* corresponding to each composition using equation 10 (refs 27, 28, 29, 30). As seen, *C* for this wide variety of metallic glass compositions is in the range of ∼2–4 × 10^7^ A √s m^−2^.

## Additional information

**How to cite this article**: Kaltenboeck, G. *et al*. Shaping metallic glasses by electromagnetic pulsing. *Nat. Commun.* 7:10576 doi: 10.1038/ncomms10576 (2016).

## Figures and Tables

**Table 1:** Data for five metallic glass compositions for estimating constant *C* using Equation (10).

Metallic glass composition	Δ*T*_g_ (K)	*v*_m_ (m^3^ mol^−1^)	*c*_p_ (J m^−3^ K^−1^)	*C* (A √s m^−2^)	Refs
Au_49_Cu_26.9_Ag_5.5_Pd_2.3_Si_16.3_	103	9.23 × 10^−6^	3.79 × 10^6^	1.83 × 10^7^	27
Pt_57.5_Cu_14.7_Ni_5.3_P_22.5_	210	8.75 × 10^−6^	4.00 × 10^6^	2.68 × 10^7^	28
Pd_40_Ni_10_Cu_30_P_20_	295	7.92 × 10^−6^	4.42 × 10^6^	3.34 × 10^7^	29
Zr_41.2_Ti_13.8_Cu_12.5_Ni_10_Be_22.5_	320	9.95 × 10^−6^	3.52 × 10^6^	3.11 × 10^7^	29
Fe_70_Ni_5_Mo_5_C_5_B_2.5_P_12.5_	398	6.89 × 10^−6^	5.08 × 10^6^	4.16 × 10^7^	30
